# Isolated muscle metastasis revealing colorectal cancer recurrence

**DOI:** 10.1093/omcr/omag064

**Published:** 2026-05-24

**Authors:** Joel Bondjeke Nkumindamu, Leila Afani, Othmane Zouiten, Mohamed El Fadli, Rhizlane Belbaraka

**Affiliations:** Medical Oncology Department, University Hospital Mohammed VI, Cadi Ayyad University, Faculty of Medicine and Pharmacy, Marrakesh, Morocco; Medical Oncology Department, University Hospital Mohammed VI, Cadi Ayyad University, Faculty of Medicine and Pharmacy, Marrakesh, Morocco; Medical Oncology Department, University Hospital Mohammed VI, Cadi Ayyad University, Faculty of Medicine and Pharmacy, Marrakesh, Morocco; Medical Oncology Department, University Hospital Mohammed VI, Cadi Ayyad University, Faculty of Medicine and Pharmacy, Marrakesh, Morocco; Medical Oncology Department, University Hospital Mohammed VI, Cadi Ayyad University, Faculty of Medicine and Pharmacy, Marrakesh, Morocco

**Keywords:** skeletal muscle metastasis, abdominal muscle, colorectal cancer, adenocarcinoma

## Abstract

Colorectal cancer is the first digestive cancer in terms of incidence, and the most common metastatic sites are the liver, lungs and peritoneum. Skeletal muscle metastases are exceptional, accounting for less than 1% of cases. Their rarity may be explained both by the biological characteristics of skeletal muscle and by the frequent underdiagnosis of this unusual metastatic site. We report here the case of a 68-year-old woman diagnosed with sigmoid adenocarcinoma who underwent immediate surgery with recto-sigmoid resection followed by adjuvant chemotherapy. Two years later, she presented with two painless abdominal masses, one located in the right flank and the other in the left subumbilical region. Thoraco-abdominopelvic CT revelated two intra-muscular lesions in the rectus abdominis muscle. No other metastatic sites were detected. Abdominal MRI confirmed the suspicious nature of the muscle masses, and the diagnosis of muscle metastases of colorectal origin was confirmed by biopsy.

## Introduction

Colorectal cancer (CRC) represents the third most prevalent malignancy worldwide and ranks as the second cause of cancer-related mortality.

The liver, lung and peritoneum are the most common metastatic sites, whereas skeletal muscle metastasis (SMM) is extremely rare, with only 30 cases reported in the English literature [1].

We report a rare case of an isolated recurrence arising the rectus abdominis muscle, detected two years after curative treatment for colon adenocarcinoma.

## Case presentation

A 68-year-old woman presented in July 2021 with acute bowel obstruction, a rectosigmoid resection was promptly performed.

Histopathology revealed a well-differentiated and infiltrating grade 1 adenocarcinoma; measuring 3 cm, extending to the subserosa without breaching it. Vascular emboli were present, with no perineural invasion.

Surgical margins were negative, including a circumferential resection margin of 2 cm. Lymph node dissection showed 0/6 nodes positive. pT3N0Mx (TNM 2017).

No secondary lesions detected on the thoracoabdominal-pelvic CT (TAP CT).

CEA: 1.05 ng/ml. CA 19–9: 5.93 U/ml.

She was classified as stage II high-risk; the case was discussed at a multidisciplinary tumor board (MTB) and adjuvant chemotherapy was recommended, 6 months of FOLFOX (fluorouracil, leucovorin and oxaliplatin).

**Figure 1:**
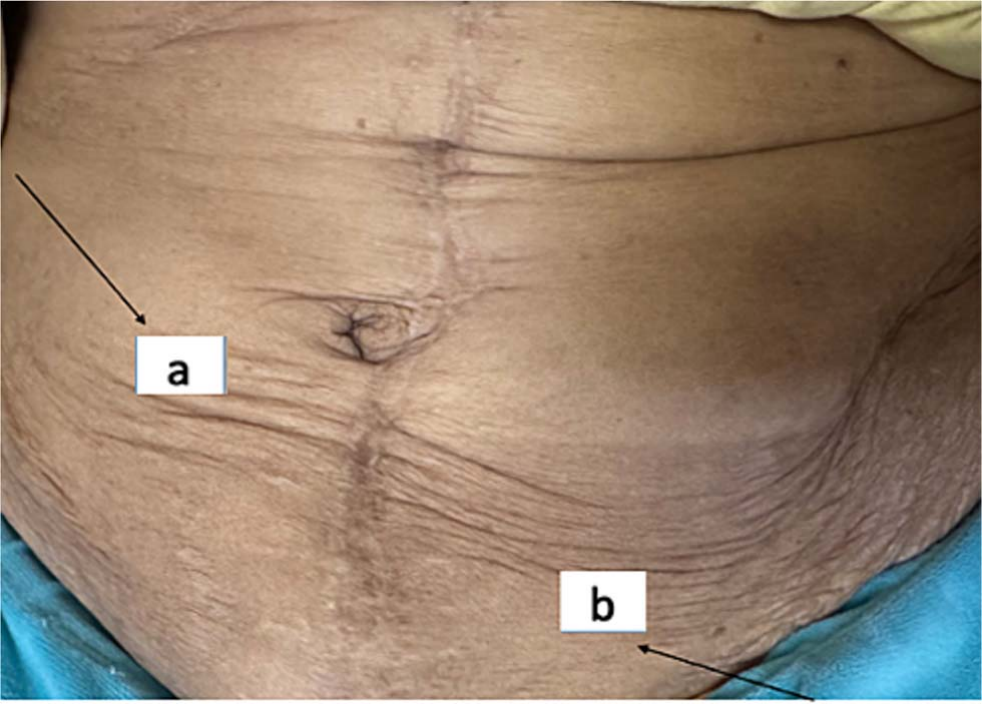
Right flank nodule (a) and left subumbilical nodule (b).

Two years later, she developed two painless abdominal nodules: measuring 4-cm in the right flank and 6-cm in the left subumbilical region (Fig. 1).

**Figure 2:**
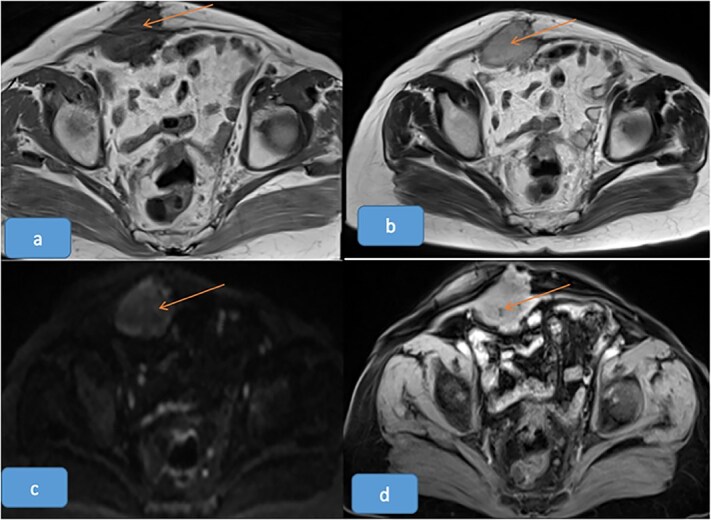
Right-sided anterior abdominal wall lesion showing T1 hypointensity (a), intermediate T2 signal (b), mild diffusion hyperintensity (c), and post-contrast enhancement (d).

**Figure 3:**
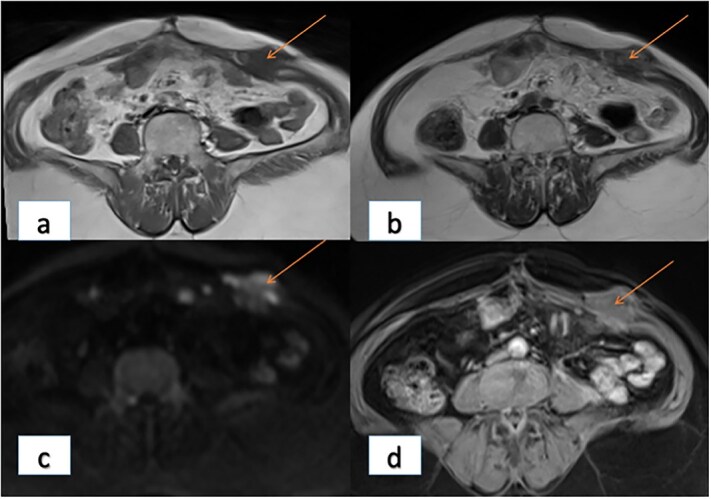
Left-sided anterior abdominal wall lesion with T1 hypointensity (a), intermediate T2 signal (b), mild diffusion hyperintensity (c), and post-contrast enhancement (d).

Magnetic resonance imaging (MRI) of abdomen characterized two parietal masses with cystic and fleshy components, with intra-abdominal development: right paramedian mass of 6.3 × 4 × 6 cm (Fig. 2) and left of 6 × 3 × 3 cm (Fig. 3); with a suspicious appearance.

No other metastatic localizations on the TAP CT.

**Figure 4:**
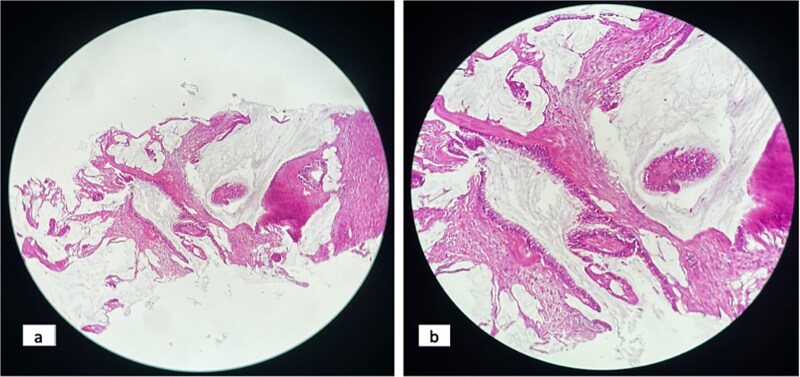
Hematoxylin and eosin staining: Poorly differentiated, infiltrative carcinoma. X 10 magnification (a); x 20 magnification (b).

**Figure 5:**
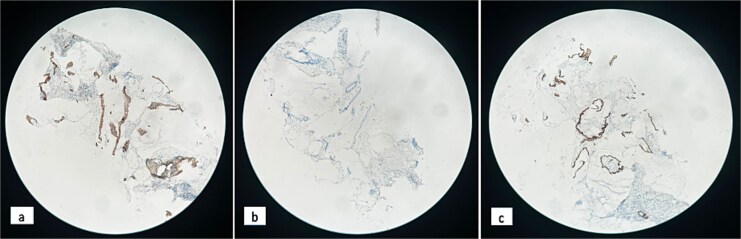
Immunohistochemistry, x 10 magnification: Cytoplasmic CK20 positivity (a), absence of CK7 expression (b), nuclear CDX2 positivity (c).

Biopsy revealed poorly differentiated carcinomatous proliferation (Fig. 4). Immunohistochemistry revealed a poorly differentiated carcinoma, expressing CK20 and CDX2 but negative for CK7, an immunophenotypic profile suggesting a CRC origin (Fig. 5).

This was an isolated metastatic relapse in skeletal muscle of a colonic adenocarcinoma.

The case was discussed at MTB, surgical resection was not retained due to multiplicity, tumor size, absence of symptoms, and the patient’s advanced age, with an Eastern Cooperative Oncology Groupe Performance Status (ECOG PS) of 2. Palliative chemotherapy with FOLFOX was indicated. KRAS and BRAF mutation testing and MSI status were ordered.

The first cycle was complicated by grade 4 gastrointestinal toxicity. The patient was lost to follow-up for two months and returned with an ECOG PS of 3 and newly developed third subumbilical mass. She has since refused any further treatment or assessment.

## Discussion

Although skeletal muscle constitutes nearly half of total body mass, metastatic spread to skeletal muscle is exceptionally rare, accounting for fewer than 1% of hematogenous metastases [2]. The possible mechanism of dissemination to skeletal muscles could be by lymphatics, hematogenous route, or direct extension of primary disease, and by manipulation during surgery [3].

Tuoheti et al. reported an incidence of 0.16% based on CT scan, with lung cancer being the primary tumor most frequently linked to SMM [4].

The thigh muscles are the most frequently affected sites [1].

SMM is rarely isolated. They most often presents as multiple lesion or in association with other metastatic sites [5]. In a large series by Cha et al. involving 71 patients with SMM, only 6 patients had isolated skeletal muscle involvement without evidence of additional metastatic sites [6].

In our case, the patient presented two isolated metastatic muscle lesions, with no evidence of other metastatic sites.

A literature review identified 30 cases of CRC metastatic to muscle between 1970 and 2022, with only one case involving the rectus abdominis muscle, a single, isolated lesion (Table 1) [1].

**Table 1:** Reports of skeletal muscle metastases in the literature. Table summarizing previously published cases, adapted from [1].

Case	Reference	Age/sex	Site of primary tumour	Presenting symptom	Site of skeletal metastasis and treatment	Non-skeletal metastases	Time Interval in mo	Follow-up/outcome
1	Hasegawa et al, 2000	60/M	Transvers colon	Not described	Right extensor carpi ulnaris muscle; a major part of the right extensor carpi ulnaris and the extensor digiti minimi muscle were resected, warranting a sufficient margin of 5 cm of normal tissue from the tumour	Multiple hepatic metastases detected 14 mo after primary resection and was resected	24	Alive
2	Buemi et al, 2019	69/F	Right colon	Pain when mobilizing left leg + elevated CEA of 7.7 ng/mL	Left gluteus muscle; lesion was resected en bloc	None	7	Alive; 6 yr after colectomy and 65 mo after resection of the muscular metastasis she was tumour free with normal CEA level
3	Yi et al, 2015	67/M	Caecum	Swelling and pain	Right thenar muscles; treatment was given with palliative systemic chemotherapy (FOLFIRI)	Liver, right kidney, right abdominal wall, left axillary and right subclavicular lymph nodes, skin of right thigh	Synchronous	Dead (9 mo after diagnosis)
4	Araki et al, 1994	66/M	Ascending colon	Painful lump	Right teres major; excision of the mass was performed		6	Dead (31 mo after surgery)
5	Manafi-Farid et al, 2019	23/M	Rectum	Incidentally detected in FDG-PET studies	Multiple: Deltoid, external oblique, biceps, tongue; excisional biopsy of the deltoid muscle lesion proved to be metastatic adenocar- cinoma; commenced chemotherapy (FOLFIRI)	Lung/adrenal gland/scalp	24	Alive
6	Torosian et al, 1987	69/M	Transverse colon	Not described	Left thigh; bloc resection was performed		60	Not specified
7	Okada et al, 2009	70/M	Rectum	Painful lump	Right thigh; resection and chemotherapy were given	Lung	12	Alive; the resection of SMM made a positive contribution to his quality of life
8	Chang et al, 1994	62/M	Descending colon	Painful lump	Left tibialis anterior; excision of the mass was performed		Synchronous	Not specified
9	Yoshikawa et al, 1999	54/M	Sigmoid colon	Severe buttocks pain	Right buttocks; en bloc resection performed	Multiple metastases	24	Died after 8 mo from multiple metastases
10	Guo et al, 2021	43/M	Ascending colon	Right thigh mass 4 cm × 4 cm with intolerable pain	Right thigh; a complete resection was suggested but was refused by the patient; unresponsive to FOLFIRI; switched to bevacizumab, irinotecan, and capecitabine	Bony metastasis and multiple lymph node metastases around the abdominal aorta	5	Deteriorated and died 9 mo after primary resection
11	Tatsuta et al, 2022	83/M	Ascending colon	Pain in the back of his neck	Cervical (neck muscle); he was prescribed palliative radiation therapy because of his poor performance status	None	11	Died 2 mo after diagnosis of muscle metastasis
12	Iusco et al, 2005	73/F	Ascending colon	Painful lump	Left calf; the mass was excised and received adjuvant radiotherapy	None	24	Alive; no sign of recurrence at a 2-yr follow-up
13	Landriscina et al, 2013	71/F	Right colon	Detected on PET/CT scan	Deltoid, sternocleidomastoid and other multiple sites; chemotherapy with FOLFOX was administered for 3 cycles but discontinued due to traumatic femur fracture	Liver/lung	23	Disease progression and death
14	Hattori et al, 2008	64/F	Rectum	Asymptomatic; increased CEA; discovered by FDG-PET	Right thoracic paraspinal muscles; en bloc excision was performed including the paraspinal muscles	Solitary lung metastasis, which was resected 3 yr previously by lobectomy with subsequent immunochemotherapy	96	Alive
15	Choi et al, 2008	83/F	Rectum	Painful lump	Semimembranous muscle of right thigh	Solitary pulmonary nodule in left lobe	48	Died of heart failure on second postoperative day
16	Doroudinia et al, 2019	48/M	Rectum	Subcutaneous lump	Right proximal thigh; the patient became a candidate for tumour excision (metastasectomy) followed by additional course of chemotherapy.	None	38	Not specified
17	Tunio et al, 2013	28/M	Ascending colon	Abdominal pain and hard nodule at anterior abdominal wall	Rectus abdominis muscle and right gluteus maximus; underwent palliative radiotherapy followed by systemic chemotherapy	None	11	Alive at time of publication with progressive disease
18	Simeunovic et al, 2014	55/F	Rectum	Lower back pain and left hip pain as first manifestation of the primary tumour	Left adductor muscle	None	Synchronous	Not specified
19	Prabhu et al, 2017	69/M	Rectum	Severe low back ache	Multiple skeletal muscles: left sartorious, left vastus lateralis, left infraspinatus, left levator scapulae, left tenth Intercostal muscle, right subscapularis muscle	None	4	Not specified
20	Tai et al, 2014	81/M	Caecum	Severe right shoulder pain	Right supraspinatus muscle	Right lobe of lung	Synchronous	Patient transitioned to hospice
21	Farraj et al, 2021	52/F	Rectum	Noted with preoperative staging	Left psoas muscle	None	Synchronous	Patient is currently maintained on platinum doublet chemotherapy with control of metastatic disease
22	Salar et al, 2012	67/F	Rectum	Deep pelvic and left buttock pain	Left piriformis muscle	None	18	Patient began cycles of chemoradiotherapy with plans for further surgical resection
23	Homan et al, 2000	72/F	Descending colon		Thigh			NA
24	Takada et al, 2011	71/M	Sigmoid colon		Left iliopsoas muscle; received radiotherapy and 15 courses of FOLFOX + bevacizumab for decreasing large and unresectable tumour; then resection was performed	GI metastasis	60	5 mo after resection of muscle metastasis, there was no recurrence
25	Naik et al, 2005	56/M	Ascending colon	A lump	Rectus abdominis muscle; resection was performed	NA	60	Not specified
26	Burgueño Montañés and López Roger, 2002	60/M	Rectosigmoid	Exophthalmos	Lateral rectus muscle			Not specified
27	García Fernández et al, 2012	32/M	Colon	Palpebral oedema, conjunctival chemosis, severe exophthalmos, complete ptosis in left eye and limitation in eye movement mainly in abduction and supraversion	Superior rectus elevator muscle of upper eyelid complex and external rectus muscle			Due to the patient generally feeling unwell, radiotherapy was not considered, and an intravenous bolus of corticoids was given, without response, resulting in the death of the patient
28	Lampenfeld et al, 1990	75/F	Rectum	Progressive growth of left buttock mass	Left gluteus maximus and medius		24	
29	Laurence and Murray, 1970; Case 1	70/F	Caecum	Painful mass in poster-oexternal aspect of right calf and leg	Right calf; en bloc resection was performed	Generalized metastasis	24	Died due to generalized metastasis
30	Laurence and Murray, 1970; Case 2	51/M	Transverse colon	oedema	Right forearm; en bloc resection was performed	Generalized metastasis	Synchronous	Died due to generalized metastasis

The apparent rarity of SMM likely reflects under-recognition, frequent absence of symptoms and the limited sensitivity of routine imaging.

Garcia et al. reported a series of autopsies in 194 patients with malignant tumors, 17.5% of malignant tumors exhibited either macroscopic and microscopic metastases in skeletal muscle suggesting higher true prevalence than clinically detected [7].

The rarity of muscle metastases may be related to the unique structural and cellular characteristics of skeletal muscle, as well as to immune factors, with a strengthened local antitumor immune response, potentially amplified by the muscle’s contractile activity. Features of the skeletal muscle microenvironment, including mediators such as myokine, may reduce tumor risk [8].

The potential antitumor effect of contractile activity is supported by Weiss et al. who observed greater cancer cell survival in denervated muscles than in electrically stimulated muscles [9].

Clinically, SMM may present as a painful or painless mass, isolated pain syndrome, or remain asymptomatic.

In the present case, presentation consisted of two abdominal masses, without pain or other associated symptoms.

Advanced imaging techniques such as fluorodeoxyglucose-positron emission tomography has improved detection of SMM including asymptomatic lesions, thereby guiding management [3].

In this case, abdominal MRI delineated two intra-abdominal parietal masses, needle biopsy revealed a poorly differentiated carcinoma expressing CK20 and CDX2 but negative for CK7, an immunophenotypic profile most consistent with colorectal origin.

To date, there is no established standard strategy for the management of CRC with SMM; management should be individualized within an MTB. It can include observation, radiotherapy, chemotherapy, and surgical excision (Table 1) [1].

In the present case-an older patient with ECOG PS of 2 and two large asymptomatic muscle metastases-the MTB opted for systemic treatment to control tumor progression and improve quality of life.

SMM is associated with poor prognosis, with reported mean survival time from diagnosis to death of 5.4 months [10].

In the present case, after chemotherapy was discontinued due to grade 4 gastrointestinal toxicity following the first cycle, the patient experienced rapid clinical progression, with deterioration to ECOG PS 3 and the appearance of a third abdominal mass.

SMM from CRC is extremely rare and often underdiagnosed due to their atypical presentation. We report a case of an isolated metastatic relapse in the rectus abdominis muscle occurring two years after curative treatment of her colon cancer. This case highlights the importance of considering SMM in patients with CRC presenting with unusual muscular symptoms. Although prognosis remains poor, early recognition may permit individualized management focused on symptom control, quality of life, and, where possible, survival.
